# Assessing agricultural practices and insecticides resistance for effective malaria vector control in northwestern Iran

**DOI:** 10.1186/s41182-024-00653-w

**Published:** 2024-11-07

**Authors:** Madineh Abbasi, Saideh Yousefi, Fatemeh Nikpour

**Affiliations:** 1 Infectious and Tropical Diseases Research Center, Tabriz University of Medical Sciences, Tabriz, Iran; 2 Sirjan School of Medical Sciences, Sirjan, Iran; 3 Student Research Committee, Sirjan School of Medical Sciences, Sirjan, Iran; 4 Institute for Environmental Research, Tehran University of Medical Sciences, Tehran, Iran; 5 Department of Vector-Borne Diseases, Centre for Communicable Diseases Control, Ministry of Health, Tehran, Iran

**Keywords:** *Anopheles*, Insecticide resistance, Iran, Malaria

## Abstract

**Background:**

After three years with no local transmission of malaria, an outbreak occurred in Iran in 2022. Key malaria control methods in Iran are including indoor residual spraying (IRS), long-lasting insecticide-treated nets (LLINs), and prompt diagnosis and treatment of malaria cases. *Anopheles sacharovi* is one of the main malaria vectors in Iran. This study aimed to determine the insecticides resistance status of *An. sacharovi* in northwestern Iran, to inform effective vector control programs in this region.

**Methods:**

Larval stages of *An. sacharovi* were collected from various larval habitats located in the villages along the Aras River. Adult susceptibility tests were performed on *An. sacharovi* using diagnostic doses of insecticides accordance to World Health Organization (WHO) guidelines. The study also evaluated agricultural insecticide and fertilizer usage alongside the presence of natural mosquito predators in breeding sites in the study area.

**Results:**

Alongside various chemicals such as silica, humic acid, superphosphate, sulfur, urea, and solupotasse at different dose levels, organophosphorus and pyrethroid insecticides are commonly used in rice fields and orchards. *Anopheles sacharovi* displayed diverse reactions to insecticides, demonstrating resistance to DDT but sensitivity to malathion, and showing similar reactions to carbamate and pyrethroid insecticides.

**Conclusions:**

These results provide significant insights into agricultural practices and the presence of mosquito larvae in the study area. The extensive use of a specific herbicide illustrates its popularity among farmers for weed control, while other agricultural products focus on enhancing soil fertility and productivity. The absence of mosquito larvae in habitats with predators indicates the usefulness of these predators in controlling the population of mosquitoes. The resistance of mosquitoes to certain insecticides highlights the need for careful selection and intermittent use of insecticides in vector control programs. These findings can inform the development of targeted strategies to reduce malaria transmission risks. Further research is essential for assessing the effectiveness of these interventions.

**Supplementary Information:**

The online version contains supplementary material available at 10.1186/s41182-024-00653-w.

## Introduction

Over 80% of the global population resides in regions vulnerable to arthropod-borne diseases [1, 2]. Mosquito, as one of the most important vectors, is involved in the transmission of a wide variety of parasites, viruses, and bacteria affecting both humans and animals [3]. According to reports by the World Health Organization (WHO), there were an estimated 249 million new cases and 608,000 deaths of malaria globally in 2022, primarily in malaria-endemic countries [4]. Iran, as part of the WHO Eastern Mediterranean Region (EMRO), has made significant progress in malaria elimination. The country has successfully reduced malaria cases in recent years; however, outbreaks reported from southeastern Iran since 2022 have raised concerns about the risk of malaria re-emergence in other regions [5].

In 2022, the country experienced a resurgence of the disease, with approximately 1,432 locally acquired malaria cases (total cases reaching 5,677), representing a tenfold increase compared to the previous year. Furthermore, in 2023, the number of confirmed cases doubled compared to the same period in the prior year, culminating in a total of 9868 cases. This fluctuation underscores the ongoing challenges in malaria control efforts within the region [4].

As vectors of malaria, *Anopheles* mosquitoes transmit *Plasmodium* parasites and are broadly distributed across all continents [6]. In Iran, 28 definitive species, including seven main malaria vectors such as *Anopheles stephensi, Anopheles culicifacies, Anopheles sacharovi, Anopheles fluviatilis, Anopheles superpictus, Anopheles maculipennis, and Anopheles dthali* have been documented [7]. Notably, *An. sacharovi* has a more localized distribution in central, northwestern, and southwestern Iran and played a crucial role during malaria outbreaks in northwestern regions [7, 8].

In Iran, the primary control methods during the malaria elimination phase are indoor residual spraying (IRS), free distribution of long-lasting insecticide-treated nets (LLINs), and early diagnosis and prompt treatment of malaria cases [9]. 

Research suggests that continuous and excessive use of insecticides can lead to resistance development in mosquito populations over time, as prolonged exposure allows them to develop mechanisms to survive despite the presence of the chemicals [10–13].

The use of agricultural insecticides and chemical or biological fertilizers, which are natural substances containing living microorganisms and enhance soil fertility and promote plant growth, can significantly impact the resistance of mosquitoes, particularly in species such as *Anopheles arabiensis* [14, 15]. Herbicides affect the timing of larval pupation, alter adult longevity, and influence susceptibility to insecticides, underscoring the importance of herbicide management in both agriculture and malaria control efforts [16].

Traditional insecticides are expensive, cause resistance in mosquitoes and also have adverse effects on non-target organisms, but biological control methods, such as the use of fish, dragonfly nymph, copepods, and certain mosquito species, are not only cheap and and effective in eliminating the mosquito populations, but they also do not have negative effects on non-target organisms [17].

Khoda-Afarin County, currently free of local malaria transmission, experienced an outbreak in 1998 due to imported cases following the conflict between Azerbaijan and Armenia, with local transmission persisting for 15 years [18]. During the period of local transmission, various control measures, including chemical control and environmental managements, were implemented during that period. Also, agricultural practices shifted from paddy farming to cotton cultivation to reduce the breeding habitats for *Anopheles* mosquitoes [18]. Today, as there is no local transmission, agricultural practices have shifted back to paddy farming, inadvertently creating ideal breeding places for *An. sacharovi* during the hot season, which lasts for approximately 6 months in the study area [19].

Given the recent increase in malaria cases across Iran, it is crucial to understand the susceptibility status of *An. sacharovi* to various WHO-recommended insecticides, especially in areas with numerous mosquito breeding sites such as the Aras River basin. This study evaluated the resistance status of *An. sacharovi* to pyrethroids, carbamates, organochlorines, and organophosphates to inform effective vector control strategies in the area. Additionally, the research investigated the usage of pesticides, herbicides, and chemical and organic fertilizers by rice farmers, as these practices can influence mosquito resistance. Furthermore, the presence of natural larval predators in the study areas was assessed as well to explore potential integrated control measures against mosquito populations.

## Methods

### Study area

**Fig. 1:**
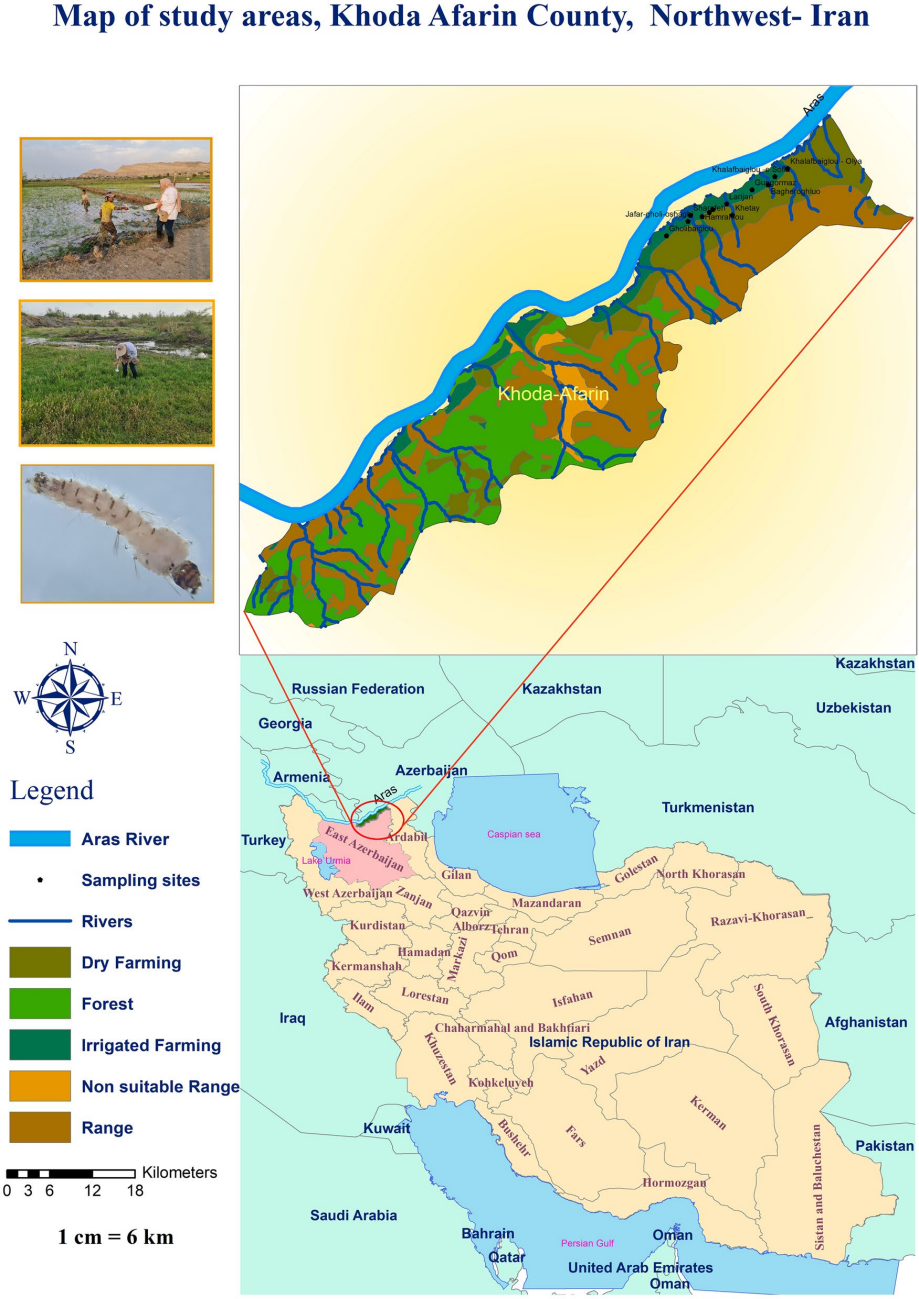
Study area, Khoda-Afarin County, northwest of Iran

Khoda-Afarin County is located in the northwest of Iran, bordered by Armenia and Azerbaijan to the north, Kaleybar County to the east, and Varzaghan and Jolfa Counties to the south and west, respectively. It is situated at approximately 38° 50′ 0" North latitude and 45° 32′ 0" East longitude. The elevation of the region ranges from 1,200 to 3,000 m above sea level and is surrounded by the Caucasus mountains, which create a unique microclimate by shielding it from cold winds. The Aras River serves as a significant natural border between Iran, Armenia, and Azerbaijan, playing a crucial role in providing irrigation, drinking water, and hydroelectric power. Additionally, the river supports diverse plant and animal life, including mosquitoes. This region experiences a continental climate with hot summers (temperatures reaching up to 35–40°^C^) and cold, snowy winters. The significant temperature fluctuations facilitate rice cultivation, that thrives due to the abundant water supply from the Aras River [20] (Fig. 1).

### Collecting the specimens

The study was conducted from June to the end of July 2023. *Anopheles sacharovi* larvae were collected from various breeding sites within 12 Aras riverside villages (Sup. 1) using a standard dipping method. All biological materials of larvae and pupae were placed in special containers previously coded and transported to the Insectarium for Species identification [21] and the lifting of the mosquito colony under standardized laboratory conditions of temperature (27 ± 2°^C^), relative humidity (75 ± 10%) and photoperiod of 12:12 (day and night), for later use in susceptibility bioassays with F1 [22].

### Agricultural practices, chemical usage, and natural predators

Simultaneously with the collection of mosquito larvae, a structured questionnaire was administered to assess agricultural practices, including the types of fertilizers, herbicides, and pesticides used. Questions were directed at farmers to inquire specifically about the types of pesticides and herbicides employed, as well as both chemical and organic fertilizers. Participants were also asked to provide samples of these consumables when possible. Additionally, interviews were conducted with local vendors of fertilizers and pesticides to confirm and supplement the information gathered from farmers.

Furthermore, during visits to each rice farm where larval collection occurred, the presence of mosquito larvae predators was also evaluated. This evaluation is critical for understanding the dynamics of mosquito population control in this region.

### Adult susceptibility tests

To assess the susceptibility of *An. sacharovi* to different insecticides, WHO guidelines for diagnostic doses were followed. Four replicate exposure tests were conducted for each insecticide, with 20–25 female mosquitoes per test. Additionally, two control tests with 50 mosquitoes were performed using papers impregnated solely with carrier oil. The insecticides tested included bendiocarb (0.1%), permethrin (0.75%), malathion (5%), DDT (4%), and deltamethrin (0.05%). Tests utilized sugar-fed F0 progeny of wild-caught adult females aged 3–5 days. Before testing, the susceptibility test kits were thoroughly cleaned with detergent and tap water. Insecticide-impregnated papers were placed in labeled tubes, alongside control tubes without insecticide for comparability [23, 24].

Mosquitoes were exposed for 60 min, followed by a 24-h recovery period with a cotton wool pad soaked in a 10% sugar solution. Mortality rates were recorded at the end of each test. Susceptibility was determined based on these rates: control mortality below 5% is acceptable, whereas rates between 5–20% were adjusted using Abbott's formula. Tests with control mortality above 20% were invalid and would be repeated. According to WHO guidelines, mortality rates of 98–100% indicated susceptibility, while rates of 90–97% flagged potential resistance, necessitating further confirmation. Rates below 90% were classified as resistant [23, 24].

## Results

Data collected from completed questionnaires regarding plants cultivated and the chemicals and organic materials used in the fields across the 12 studied villages revealed that 75% of rice farmers used bispyribac sodium herbicides, 25% used silica, 58% used humic acid, and 50% employed superphosphate, 8.3% used sulfur, 83% utilized urea, and 25% applied fertilizers. The use of organophosphorus and pyrethroid insecticides were observed in rice fields and orchards, although this was limited to a few areas. Specifically, pyrethroids, including cypermethrin (EC 40%) and deltamethrin (EC 2.5%), were reported in approximately 17% of the areas, respectively (Table 1). Notably, no mosquito larvae were found in breeding habitats that contained dragonfly nymphs or Gambusia fish, both of them are natural predators of mosquito larvae (Table 1).

**Table 1:** History of consumption of organic and chemical substances used in mosquito larval habitats in Khoda-Afarin, Iran, 2023

Village	History of organic and chemical substances use			Gambusia fish (G)/dragonfly nymph (D)	Mosquito (genus)
	Herbicide	Fertilizer	Insecticide		
Hamrahlou	Yes	Urea	No	D	Not identified
Khetay	No	No	No	None	Not identified
Larijan	Yes	Yes (silica, humic acid, superphosphate, sulfur, urea, solupotasse)	Yes (cypermethrin EC 40%, deltamethrin EC 2.5%, chlorpyrifos 40.8% EC, diazinon 8.3%)	G/D	Not identified
Parvizkhanlou	Yes	Yes (humic acid, superphosphate, urea)	Yes (cypermethrin EC 40%, diazinon 8.3%)	G/D	Not identified
Sharafeh	No	Yes (silica, superphosphate, urea)	No	G/D	Not identified
Mohammad Salehlou	Yes	Yes (humic acid, urea)	Yes (diazinon 8.3%)	D	*Culex spp.* *Anopheles spp.*
Gungormaz	Yes	Yes (humic acid, superphosphate, urea, solupotasse)	Yes (diazinon 8.3%)	G/D	Not identified
Jafar-Gholi-Ushaghi	Yes	Yes (humic acid, superphosphate, urea)	Yes (diazinon 8.3%)	G/D	Not identified
Khalafbaiglou -e Sofla	No	No	No	G/D	Not identified
Khalafbaiglou-e Olya	Yes	Yes (superphosphate, urea, solupotasse)	No	None	*Culex spp. * *Anopheles spp.*
Bagheroghluo	Yes	Yes (humic acid, urea)	No	G/D	Not identified
Gholibaiglou	Yes	Yes (silica, humic acid, urea)	No	None	*Aedes spp.* *Culiseta spp.*

D = dragonfly nymph, G = gambusia fish

The species exhibited varied reactions to organophosphorus and organochlorine insecticides, demonstrating complete resistance to DDT while being sensitive to malathion. They displayed similar responses to carbamate and pyrethroid insecticides. Specifically, bendiocarb, permethrin, and deltamethrin, tested in two repetitions, resulted in mortality rates between 97 and 98% (Table 2).

**Table 2:** Resistance status of *Anopheles sacharovi* to different insecticides in Khoda-Afarin County, Northwest Iran, 2023

Insecticide	Test Repetition	Exposure time (H)*	Trial test			Control		
			Total (N)**	Dead (N)	Mortality (%) ± SD***	Total (N)	Dead (N)	Mortality (%) ± SD
DDT 4%	2	1	259	223	86.10 ± 3.61	70	2	2/85 ± 0.5
Permethrin 0.75%	1	1	98	96	97/96 ± 5.2	52	0	0 ± 0
Bendiocarb 0.1%	1	1	145	142	97/93 ± 2.22	40	0	0 ± 0
Deltamethrin 0.05%	2	1	222	216	97.3 ± 2.34	64	2	3.12 ± 0.2
Malathion 5%	1	1	103	103	100 ± 0	35	0	0 ± 0

^*^H: hour

^**^N: number

^***^standard deviation

## Discussion

Iran launched a malaria elimination initiative in 2009, aiming to achieve certification by 2025 [4, 25]. The primary strategies for controlling and preventing malaria in the country consist of five key interventions: IRS in households, distribution of free LLINs for all people at risk of malaria, providing complimentary malaria diagnosis and treatment (active and passive case detection), conducting emergency space fogging, and manipulating or physically eliminating mosquito breeding places [26]. Therefore, insecticide-based mosquito control remains critically important in vector control strategies in this country [9].

Currently, chemical control methods against malaria vectors are limited to the southeastern regions of Iran [27]. Major challenges in these infected areas stem from the conflict in Afghanistan and the migration of a large population into various parts of Iran in 2022, alongside legal and illegal fuel trade with malaria-endemic regions of Pakistan. This migration and trade have contributed to the importation of malaria cases from neighboring countries, leading to the re-emergence of malaria in southeastern Iran. Some of these malaria reservoirs travel to other provinces of Iran where potential malaria vectors exist. therefore, health systems in these areas must be prepared to deal with possible outbreaks [28, 29].

*Anopheles sacharovi* is a potential vector of malaria in northwestern Iran, the anthropophily index of this species is notably high (38.5%), and this species was identified as a crucial vector during malaria outbreaks in this region [8].

The current research assesses the susceptibility of *An. sacharovi* to various insecticides, including DDT, permethrin and deltamethrin, malathion, and bendiocarb in Khoda-Afarin County, situated in East Azerbaijan Province, northwestern Iran. Although pesticides such as cypermethrin EC 40% and deltamethrin EC 2.5% (pyrethroids), along with chlorpyrifos EC 40.8% and diazinon 8.3% (organophosphates), are employed in rice fields for agricultural pests control, but *An. sacharovi* remains sensitive to permethrin, bendiocarb, and malathion, which belong to the pyrethroid, carbamate, and organophosphate categories, respectively. Notably, this species shows complete resistance to DDT in the study area.

Monitoring of insecticide resistance in *An. sacharovi* has been conducted in various regions of northwestern and southern Iran over the years. In the work carried out, it was reported that in East Azerbaijan Province (Kaleybar County that Khoda-Afarin was part of this county historically) at 19 years ago, *An.‌sacharovi* exhibited resistance to DDT, tolerance to dieldrin, and susceptibility to malathion, permethrin, and deltamethrin [30]. In another investigation in this area, the susceptibility of *An.‌sacharovi* to several insecticides (DDT 4%, malathion 5%, permethrin 0.75%, dieldrin 0.4%, fenitrothion 1%, and deltamethrin 0.05%) was assessed, yielding similar results of resistance to DDT and tolerance to dieldrin, while demonstrating susceptibility to the rest [31].

A study performed in West Azerbaijan Province, bordering our study area, indicated that *An.‌sacharovi* was resistant to DDT and tolerant to dieldrin, while remaining susceptible to bendiocarb, cyfluthrin, deltamethrin, fenitrothion, lambdacyhalothrin, permethrin, malathion, propoxur, and etofenprox [32]. The findings of our research align with those of other studies in East and West Azerbaijan Provinces, showing that *An.‌sacharovi* is resistant to DDT and sensitive to permethrin, deltamethrin, malathion, and bendiocarb. Unfortunately, due to the unavailability of impregnated insecticide papers, we could not assess the resistance status of *An. sacharovi* to dieldrin.

In Ardabil Province, located to the northwest of Iran and adjacent to our study area, *An.‌sacharovi* was resistant to DDT [8], tolerant to permethrin 0.25% and deltamethrin 0.025%, and susceptible to malathion 5% and propoxur 0.1% [27]. Other studies revealed that, this species was resistant to both DDT and dieldrin, alongside sensitivity to malathion, lambdacyhalothrin, propoxur, deltamethrin, cyfluthrin, and bendiocarb in Ardabil Province [8, 32].

Our research aligns with findings from Ardabil Province, indicating that *An. sacharovi* exhibits resistance to DDT [8] while remaining sensitive to malathion and bendiocarb. However, in Ardabil Province this species showed tolerance to permethrin and deltamethrin. It seems that the reason for these differences is the widespread use of insecticides to control the vectors of zoonotic visceral Leishmaniasis in Ardabil province while such an operation is not carried out in the studied area.

In Fars Province, located in southwestern Iran, *An.‌sacharovi* is susceptible to DDT 4% and resistant to fenitrothion 1% [33]. The varying environmental conditions between the southern and northwestern regions of Iran can influence vector ecology and subsequently interactions with insecticides.

*Anopheles sacharovi* is a primary malaria vector in Turkey, particularly along Iran's northwest border [34]. In Turkey, its susceptibility to various insecticide results indicated that in regions like Adana, Antalya, and Adiyaman, this species was susceptible to malathion and pirimiphos-methyl. In Aydin, it was also susceptible to dieldrin, lambdacyhalothrin, and etofenprox, while in Mugla it was sensitive to nearly all insecticides tested, except for propoxur, bendiocarb, permethrin, deltamethrin, and DDT [35]. Unlike our findings, *An. sacharovi* in Turkey shows resistance to permethrin, deltamethrin, and bendiocarb. The differences may stem from the wide distribution of *An. sacharovi* in Turkey, exposing it to various insecticide groups used for both health and agricultural purposes over extended periods [35].

Present study found use of various herbicides and fertilizers, including silica, humic acid, superphosphate, sulfur, urea, and solopotas, as well as pesticides such as cypermethrin EC 40%, deltamethrin EC 2.5%, chlorpyrifos 40.8% EC, and diazinon 8.3%, in rice fields of the studied region. The application of these substances varies across different areas. However, we lacked the capacity to laboratory test each of these products on *An. sacharovi*.

The application of insecticides, as well as biological and chemical fertilizers in agricultural practices significantly impacts the resistance of *Anopheles* mosquitoes [36]. Research in Africa has revealed that in regions with high insecticide usage, resistance to commonly employed insecticides, including pyrethroids, has increased. This resistance diminishes the effectiveness of LLITs and IRS, which are crucial for malaria control strategies [37]. Another study evaluated the effects of chemical and biological fertilizers on mosquito populations, finding that chemical fertilizers heightened the abundance of mosquito larvae in rice fields, thus potentially increasing malaria transmission risks [36, 38]. Conversely, biological fertilizers such as organic matter or compost did not substantially affect mosquito numbers. While agricultural practices influence mosquito populations, they are just one several factors contributing to malaria transmission [36, 39] and other critical factors include climate, human behavior, and healthcare access [11–13]. A Kenyan study also linked the use of chemical fertilizers to increased resistance to pyrethroid insecticides in *Anopheles* mosquitoes [40].

In our study area, no mosquito larvae were caught in the habitats containing Gambusia fish, and the abundance of mosquito larvae was very low in the habitats with dragonfly nymphs. The use of Gambusia fish is effective in controlling the larvae of malaria vectors, and other mosquito genera [41, 42]. For example, a study illustrated that introducing Gambusia into water bodies notably reduced *Anopheles* mosquito populations, the primary malaria vector [43]. Another study in Iran demonstrated that integrating Gambusia fish with insecticide-treated bed nets significantly reduced malaria cases [44]. A study conducted in Africa showed that *Culex* mosquitoes avoid laying their egg rafts in containers containing predatory fish [45].

Research in Thailand also noted that introducing dragonflies in rice fields substantially diminished *Aedes* mosquito populations, which are the primary vectors of dengue fever [46]. In Malaysia, dragonfly presence in residential places correlated with reduced *Aedes* populations and lower dengue incidence [47]. Mosquitoes receive predator-released kairomones (PRKs) and therefore avoid laying eggs in larval habitats containing these predators. [48, 49]. These findings suggest that the chemical compounds produced by predators can serve as an environmentally friendly method for controlling mosquito populations [49].

To mitigate insecticide resistance among mosquitoes, health systems could adopt alternative control methods such as biological strategies. In this study, breeding sites with predators yielded no mosquito larvae, likely due to two reasons: direct predation by the predators and avoiding female mosquitoes from laying eggs in predator-rich breeding habitats [42]. However, natural enemies can effectively control mosquito’s larvae without side effects on environment and non-target organisms [17, 45, 50].

The findings from this study underscore the importance of ongoing insecticide resistance monitoring and the integration of sustainable control methods in malaria vector management. By demonstrating *An. sacharovi's* resistance to DDT and sensitivity to alternative insecticides, as well as the effectiveness of natural predators like Gambusia fish and dragonfly nymphs, this research supports the need for adaptive and multifaceted strategies in malaria control. Such strategies are vital for mitigating the impact of climate change, urbanization, and cross-border malaria transmission, thereby enhancing public health efforts in Iran and similar regions.

## Conclusions

This study examines the susceptibility and resistance status of *Anopheles sacharovi* to various insecticides in East Azerbaijan Province, Iran. It highlights the complete resistance of this mosquito species to DDT while retaining sensitivity to malathion, bendiocarb, and certain pyrethroids. These findings underscore the urgent need for innovative malaria control strategies. Understanding the impact of agricultural pesticides and mosquito behavior is crucial for addressing management challenges. To effectively reduce mosquito populations and prevent the spread of vector-borne diseases, new control methods, particularly the integration of natural predators like Gambusia fish and dragonfly nymphs, should be emphasized. Continuous monitoring and research into resistance mechanisms are essential for enhancing public health initiatives against malaria and other mosquito-borne diseases.

## Supplementary Information

Supplementary material 1.

## Data Availability

All data on which this article is based are included within the article.

## Abbreviations

*An**.*: *Anopheles*
DDT: Dichloro-Diphenyl-Trichloroethane
EC: Emulsifiable Concentrate
EMRO: Eastern Mediterranean Region
H: Hour
IRS: Indoor residual spraying
PRKs: Predator-Released Kairomones
LLINs: Long-lasting Insecticide-Treated Nets
N: Sample size
SD: Standard Deviation
WHO: World Health Organization
%: Percentage

## References

[1] WHO. Global vector control response 2017–203. Geneva: World Health Organization; 2017.

[2] Vieira RFC, Muñoz-Leal S, Faulkner G, Şuleşco T, André MR, Pesapane R. Global climate change impacts on vector ecology and vector-borne diseases. In: McNabb SJN, Shaikh AT, Haley CJ, editors. Modernizing global health security to prevent, detect, and respond. Amsterdam: Academic Press; 2024. p. 155–73.

[3] Abdullah M, Hind I, Al K, Ahmed S, El-Hadeeti K, Elhadeeti SAK. The role of mosquitoes in transmitting some pathogens. 2022.

[4] WHO. World malaria report 2023. Geneva: World Health Organization; 2023.

[5] WHO. World malaria report 2022. Geneva: World Health Organization; 2022.

[6] Abbasi M, Oshaghi MA, Sedaghat MM, Hazratian T, Foroushani AR, Jafari-Koshki T, et al. Development of a degree-day model to predict the growth of *Anopheles stephensi* (Diptera: Culicidae): implication for vector control management. Environ Entomol. 2023;52:1126–1138. 10.1093/ee/nvad092 37738476 10.1093/ee/nvad092

[7] Abbasi M, Foroushani AR, Jafari-Koshki T, Pakdad K, Vatandoost H, & Hanafi-Bojd AA. The impact of climatic variables on the population dynamics of the main malaria vector, *Anopheles stephensi* Liston (Diptera: Culicidae), in southern Iran. Asian Pac J Trop Med. 2020;13(10):448–455. 10.4103/1995-7645.291038.

[8] Yaghoobi-Ershadi MR, Namazi J, Piazak N. Bionomics of *Anopheles sacharovi* in Ardebil province, northwestern Iran during a larval control program. Acta Trop. 2001;78(3):207–15. 10.1016/s0001-706x(01)00080-8. 11311184 10.1016/s0001-706x(01)00080-8

[9] Abbasi M, Hanafi-Bojd, A, Yaghoobi-Ershadi MR, Vatandoost H, Oshaghi MA, Hazratian T et al. Resistance status of the main malaria vector, *Anopheles stephensi* Liston (Diptera: Culicidae), to insecticides in a malaria endemic area, southern Iran. Asian Pac J Trop Med. 2019;12(1):43–48. 10.4103/1995-7645.250344.

[10] Nkya TE, Poupardin R, Laporte F, Akhouayri I, Mosha F, Magesa S, et al. Impact of agriculture on the selection of insecticide resistance in the malaria vector *Anopheles gambiae*: a multigenerational study in controlled conditions. Parasit Vectors. 2014;7:480. 10.1186/s13071-014-0480-z. 25318645 10.1186/s13071-014-0480-z PMC4201709

[11] Fillinger U, Sonye G, Killeen GF, Knols BG, Becker N. The practical importance of permanent and semipermanent habitats for controlling aquatic stages of* Anopheles gambiae**sensu lato* mosquitoes: operational observations from a rural town in western Kenya. Trop Med Int Health. 2004;9(12):1274–89. 10.1111/j.1365-3156.2004.01335.x. 15598259 10.1111/j.1365-3156.2004.01335.x

[12] Tusting LS, Thwing J, Sinclair D, Fillinger U, Gimnig J, Bonner KE, et al. Mosquito larval source management for controlling malaria. Cochrane Database Syst Rev. 2013;2013(8):Cd008923. 10.1002/14651858.CD008923. 23986463 10.1002/14651858.CD008923.pub2 PMC4669681

[13] Ranson H, N’Guessan R, Lines J, Moiroux N, Nkuni Z, Corbel V. Pyrethroid resistance in African anopheline mosquitoes: what are the implications for malaria control? Trends Parasitol. 2011;27(2):91–8. 10.1016/j.pt.2010.08.004. 20843745 10.1016/j.pt.2010.08.004

[14] Kouadio FA, Wipf NC, Nygble AS, Fodjo BK, Sadia CG, Vontas J, et al. Relationship between insecticide resistance profiles in *Anopheles gambiae sensu lato* and agricultural practices in Côte d’Ivoire. Parasit Vectors. 2023;16(1):270. 10.1186/s13071-023-05876-0. 37559080 10.1186/s13071-023-05876-0 PMC10410919

[15] Jeanrenaud A, Brooke BD, Oliver SV. The effects of larval organic fertiliser exposure on the larval development, adult longevity and insecticide tolerance of zoophilic members of the *Anopheles gambiae* complex (Diptera: Culicidae). PLoS ONE. 2019;14(4): e0215552. 10.1371/journal.pone.0215552. 30998732 10.1371/journal.pone.0215552 PMC6472872

[16] Oliver SV, Brooke BD. The effect of commercial herbicide exposure on the life history and insecticide resistance phenotypes of the major malaria vector Anopheles arabiensis (Diptera: culicidae). Acta Trop. 2018;188:152–60. 10.1016/j.actatropica.2018.08.030. 30179608 10.1016/j.actatropica.2018.08.030

[17] Benelli G, Jeffries CL, Walker T. Biological control of mosquito vectors: past, present, and future. Insects. 2016. 10.3390/insects7040052. 27706105 10.3390/insects7040052 PMC5198200

[18] Sarafraz S, Ghabouli Mehrabani N, Mirzaei Y, Jafari R, Ghabouli Mehrabani R, Rahnamaye Hayati V, et al. Epidemiology of malaria in East Azerbaijan province, Iran, from 2001 to 2013. J Parasit Dis. 2016;40(3):813–7. 10.1007/s12639-014-0584-6. 27605789 10.1007/s12639-014-0584-6 PMC4996197

[19] Schapira A, Zaim M, Raeisi A, Ranjbar M, Kolifarhood G, Nikpour F, et al. History of the successful struggle against malaria in the Islamic Republic of Iran—from the earliest records to imminent elimination. 2018.

[20] Data TGHWaC. Khoda Afarin, East Azarbaijan, Iran climate 2024. https://weatherandclimate.com/iran/east-azarbaijan/khoda-afarin.

[21] Azari-Hamidian S, Harbach R. Keys to the adult females and fourth-instar larvae of the mosquitoes of Iran (Diptera: Culicidae). Zootaxa. 2009;2078:1–33. 10.5281/zenodo.187282.

[22] Waldetensai A. Protocol for *Anopheles* mosquito Rearing and handling. 2020.

[23] WHO. Test procedures for insecticide resistance monitoring in malaria vector mosquitoes. 2nd ed. Geneva: World Health Organization; 2016.

[24] WHO. Manual for monitoring insecticide resistance in mosquito vectors and selecting appropriate interventions. Geneva: World Health Organization; 2022.

[25] WHO. Malaria elimination: a field manual for low and moderate endemic countries. Geneva: World Health Organization; 2007.

[26] Nikpour F, Vatandoost H, Hanafi-Bojd AA, Raeisi A, Mirolyaie A, Mojahedi AR, et al. Long-lasting residual efficacy of Actellic®300CS and Icon®10CS on different surfaces against *Anopheles stephensi*, an invasive malaria vector. Trop Med Int Health. 2024;29(9):781–91. 10.1111/tmi.14028. 39081142 10.1111/tmi.14028

[27] Vatandoost H, Hanafi-Bojd AA, Nikpour F. Monitoring and mapping of insecticide resistance in malaria vector, *Anopheles sacharovi* in iran (1997–2020). Acta Sci Malaysia. 2021;5:75–9. 10.26480/asm.02.2021.75.79.

[28] Raiesi A, Nejati J, Ansari-Moghaddam A, Sakeni M, Faraji L, Paktinat B, et al. Effects of foreign immigrants on malaria situation in cleared up and potential foci in one of the highest malaria burden district of southern Iran. Malaria J. 2012. 10.1186/1475-2875-11-S1-P81.

[29] Hemami MR, Sari AA, Raeisi A, Vatandoost H, Majdzadeh R. Malaria elimination in iran, importance and challenges. Int J Prev Med. 2013;4(1):88–94. 23413116 PMC3570917

[30] Vatandoost H, Boonab R, Abai MR, Oshaghi M, Rassi Y, Gholizadeh S. Entomological survey in Kalibar, a rurgent malaria focus in East-Azerbaijan. Iran Pakistan J Biol Sci. 2005;8:466–71.

[31] Vatandoost H. Irritability of malaria vector, *Anopheles sacharovi* to different insecticides in a malaria–prone area. Asian Pac J Trop Med. 2012;5:113–6. 10.1016/S1995-7645(12)60007-8. 22221753 10.1016/S1995-7645(12)60007-8

[32] Lak S, Vatandoost H, Entezarmahdi MR, Ashraf H, Nazari M. Monitoring of Insecticide resistance in *Anopheles Sacharovi* (Favre, 1903) in Borderline of Iran, Armenia, Naxcivan and Turkey, 2001. Iranian J Publ Health. 2002;31:96–9.

[33] Ladonni SGH. Determination of susceptibility of *Anopheles sacharovi* collected from dasht argen in Fars province to DDT, malathion fenitrothion (Diptera, Culicidae). Iran J Public Health. 1970;28(1–4).

[34] Alten B, Caglar SS, Simsek FM, Kaynas S. Effect of insecticide-treated bednets for malaria control in Southeast Anatolia-Turkey. J Vector Ecol. 2003;28(1):97–107. 12831134

[35] Kasap H, Kasap M, Alptekin D, Lüleyap U, Herath PR. Insecticide resistance in Anopheles sacharovi Favre in southern Turkey. Bull World Health Organ. 2000;78(5):687–92. 10859863 PMC2560765

[36] Kibuthu TW, Njenga SM, Mbugua AK, Muturi EJ. Agricultural chemicals: life changer for mosquito vectors in agricultural landscapes? Parasit Vectors. 2016;9(1):500. 10.1186/s13071-016-1788-7. 27624456 10.1186/s13071-016-1788-7 PMC5022241

[37] Hien AS, Soma DD, Hema O, Bayili B, Namountougou M, Gnankiné O, et al. Evidence that agricultural use of pesticides selects pyrethroid resistance within* Anopheles gambiae s.l.* populations from cotton growing areas in Burkina Faso, West Africa. PLoS One. 2017;12(3):e0173098. 10.1371/journal.pone.0173098. 28253316 10.1371/journal.pone.0173098 PMC5333875

[38] Darriet F. Synergistic effect of fertilizer and plant material combinations on the development of* Aedes aegypti* (Diptera: Culicidae) and *Anopheles gambiae* (Diptera: Culicidae) mosquitoes. J Med Entomol. 2018;55(2):496–500. 10.1093/jme/tjx231. 29309617 10.1093/jme/tjx231

[39] Victor TJ, Reuben R. Effects of organic and inorganic fertilisers on mosquito populations in rice fields of southern India. Med Vet Entomol. 2000;14(4):361–8. 10.1046/j.1365-2915.2000.00255.x. 11129699 10.1046/j.1365-2915.2000.00255.x

[40] Ochomo E, Subramaniam K, Kemei B, Rippon E, Bayoh NM, Kamau L, et al. Presence of the knockdown resistance mutation, Vgsc-1014F in *Anopheles gambiae* and *An. arabiensis* in western Kenya. Parasit Vectors. 2015;8:616. 10.1186/s13071-015-1223-5. 26626424 10.1186/s13071-015-1223-5 PMC4666190

[41] Arijo A, Ahmad L, Sethar A, Muhammad F, Bhutto B, Leghari I, et al. Biological control of mosquito larvae using edible fish. 2017. https://journalijiar.com/article/549/biological-control-of-mosquito-larvae-using-edible-fish/.

[42] Singh H, Gupta SK, Vikram K, Saxena R, Srivastava A, Nagpal BN. Sustainable control of malaria employing Gambusia fishes as biological control in Jalore and Barmer districts of Western Rajasthan. J Vector Borne Dis. 2022;59(1):91. 35708410

[43] Walshe DP, Garner P, Adeel AA, Pyke GH, Burkot TR. Larvivorous fish for preventing malaria transmission. Cochrane Database Syst Rev. 2017;12(12):Cd008090. 10.1002/14651858.CD008090.pub3. 29226959 10.1002/14651858.CD008090.pub3 PMC5741835

[44] Shahi M, Kamrani E, Shalehi M, Habibi R, Hanaf Bojd AA. Native larvivorous fish in an endemic malarious area of southern Iran, a biological alternative factor for chemical larvicides in malaria control program. Iran J Public Health . 2015;44(11):1544–9. 26744713 PMC4703235

[45] Segev O, Verster R, Weldon C. Testing the link between perceived and actual risk of predation: mosquito oviposition site selection and egg predation by native and introduced fish. J Appl Ecol. 2017;54(3):854–61. 10.1111/1365-2664.12789.

[46] Chareonviriyaphap T, Bangs MJ, Suwonkerd W, Kongmee M, Corbel V, Ngoen-Klan R. Review of insecticide resistance and behavioral avoidance of vectors of human diseases in Thailand. Parasit Vectors. 2013;6:280. 10.1186/1756-3305-6-280. 24294938 10.1186/1756-3305-6-280 PMC3850650

[47] Saleeza Ramlee S, Norma-Rashid N, Mohd SA. Odonata nymphs as potential biocontrol agents of mosquito larvae in Malaysia. Southeast Asian J Trop Med Public Health. 2022;53(4):426–35.

[48] Hegazy MI, Hegazy AM, Saad AM, Salem HM, El-Tahan AM, El-Saadony MT, et al. ٍSome biologically active microorganisms have the potential to suppress mosquito larvae (*Culex pipiens*, Diptera: Culicidae). Saudi J Biol Sci. 2022;29(4):1998–2006. 10.1016/j.sjbs.2021.12.028. 10.1016/j.sjbs.2021.12.028 PMC9072919 35531139

[49] Silberbush A, Markman S, Lewinsohn E, Bar E, Cohen JE, Blaustein L. Predator-released hydrocarbons repel oviposition by a mosquito. Ecol Lett. 2010;13(9):1129–38. 10.1111/j.1461-0248.2010.01501.x. 20618841 10.1111/j.1461-0248.2010.01501.x

[50] Huang YS, Higgs S, Vanlandingham DL. Biological control strategies for mosquito vectors of arboviruses. Insects. 2017. 10.3390/insects8010021. 28208639 10.3390/insects8010021 PMC5371949

