# Supplementary material for: Assessing agricultural practices and insecticides resistance for effective malaria vector control in northwestern Iran
Source: Trop Med Health. 2024 Nov 7;52:81. doi: 10.1186/s41182-024-00653-w (PMC11542464; doi:10.1186/s41182-024-00653-w)
Supplement: Supplementary file 1 — Supplementary material 1. [file 41182_2024_653_MOESM1_ESM.docx]

Supplementary 1. Study Villages Location Coordinates in Khoda-Afarin County, Northwest Iran

| Village | Latitude | Longitude |
| --- | --- | --- |
| Hamrahlou | 39°18'34.21"N | 47°13'19.85"E |
| Khetay | 39°17'23.45"N | 47°12'27.47"E |
| Larijan | 39°17′28″N | 47°10′13″E |
| Parvizkhanlou | 39°17′51″N | 47°10′35″E |
| Sharafeh | 39°17′15″N | 47°08′28″E |
| Mohammad Salehlou | 39°17'24.88"N | 47°10'10.99"E |
| Gungormaz | 39°19'56.73"N | 47°14'13.84"E |
| Jafar-Gholi-Ushaghi | 39°15'33.15"N | 47°09'11.52"E |
| Khalafbaiglou -e Sofla | 39°21′7″N | 47°17′42″E |
| Khalafbaiglou-e Olya | 39°20′21″N | 47°16′31″E |
| Bagheroghluo | 39°19′49″N | 47°15′49″E |
| Gholibaiglou | 39°15′36″N | 47°06′7″E |
